# Neuropsychiatric Disorders and Psychosis Induced by Chronic Toluene Exposure: Case Review and Antipsychotic Treatment Approaches

**DOI:** 10.1192/j.eurpsy.2025.1753

**Published:** 2025-08-26

**Authors:** A. Romero Teruel, A. Vives Luengo, P. Vazquez Giraldo, B. Franco Lovaco, G. Vega

**Affiliations:** 1 Psychiatry, Hospital Dr. Rodríguez Lafora; 2 Psychiatry, Hospital Universitario de La Paz; 3 Subdirección General de Humanización de la Asistencia Sanitaria, Consejería de sanidad, Madrid, Spain

## Abstract

**Introduction:**

This case of a 79-year-old patient with inferred past exposure to toluene and persistent paranoid delusions illustrates the potential neuropsychiatric consequences of solvent exposure. The patient’s work as a car painter (1990-1992) aligns with literature highlighting the neurotoxic effects of organic solvents. Despite no direct evidence of exposure, treatment with Zuclopentixol 200 mg Depot every two weeks led to symptom improvement, reflecting findings on toluene-related neuropsychiatric disorders such as psychosis.

**Objectives:**

- Present a potential case of toluene-induced psychosis.
- Review the etiopathogenesis and treatment.
- Assess evidence linking chronic solvent exposure with neuropsychiatric disorders.

**Methods:**

A literature search using PubMed databases was conducted with keywords: (toluene OR xylene OR volatile organic compounds OR organic solvents) AND (psychosis OR schizophrenia OR mental disorders). Case series and observational studies were reviewed. No randomized clinical trials on antipsychotic treatment for toluene-induced psychosis were found.

**Limitations:**

The patient’s exposure to toluene was inferred based on work history, without direct evidence such as biomarkers or occupational assessments. As most studies are case series, results must be interpreted with caution. There is a lack of randomized controlled trials exploring antipsychotic treatments in solvent-induced psychosis.

**Results:**

Chronic exposure to toluene is associated with cognitive impairment, memory deficits, personality changes, and psychosis. Neuroimaging often reveals white matter alterations and cerebral atrophy in chronic users. Rare cases of irreversible schizophreniform psychosis have been documented. Treatment with atypical antipsychotics like risperidone shows variable efficacy, but outcomes differ between patients. In this case, Zuclopentixol 200 mg Depot every two weeks led to significant symptom reduction.

**Conclusions:**

Chronic toluene exposure can result in severe neuropsychiatric disorders, including psychosis, as demonstrated by this 79-year-old patient. Neuroimaging showed cerebral atrophy and white matter changes in long-term exposure cases. Treatment with Zuclopentixol effectively reduced symptoms, despite the limited literature, which is mostly based on case series. Randomized clinical trials are needed to develop standardized treatment protocols. Additionally, occupational safety measures are critical to preventing adverse effects from solvent exposure.

**Disclosure of Interest:**

None Declared

